# Preparing the Frontline: Profiling Knowledge, Attitudes, and Practice Gaps in Healthcare‐Associated Infection Prevention Among Future Health Professionals in Belize

**DOI:** 10.1155/ipid/3745039

**Published:** 2026-06-22

**Authors:** Danladi C. Husaini, Sanjana Punjabi, Tatyana Guild, Elodia Solis, Yusuf Abubakar

**Affiliations:** ^1^ Department of Allied Health, Pharmacy Program, University of Belize, Central America, Belmopan, Belize; ^2^ Allied Health Department, Antimicrobial Resistance (AMR) Study Group, University of Belize, Faculty of Health Sciences, Belmopan Central Campus, Central America, Belmopan, Belize

**Keywords:** allied health students, Belize, curriculum development, healthcare-associated infections, infection prevention, knowledge and attitudes

## Abstract

**Background:**

Healthcare‐associated infections (HAIs) pose a significant threat to patient safety in Belize, with prevalence rates far exceeding global averages. Allied health students represent the future frontline in infection prevention, yet their preparedness in high‐burden, resource‐limited settings like Belize—where HAI prevalence approaches 40%—remains unassessed. This study aimed to conduct the first multidimensional analysis of the knowledge, attitudes, and preventive practices (KAP) regarding HAIs among allied health students at the University of Belize (UB).

**Methods:**

A descriptive cross‐sectional study was conducted among 210 randomly selected nursing, pharmacy, and medical laboratory science students, yielding an 85% response rate. Data were collected via a structured, self‐administered electronic questionnaire. Analysis included descriptive statistics, inferential tests (chi‐square, *t*‐tests, and ANOVA), multivariable regression, and latent class analysis to identify distinct student profiles.

**Results:**

The mean knowledge score was 3.84 out of 7 (54.9%), indicating moderate overall understanding. While students demonstrated strength in general transmission routes (81.9% correct) and basic prevention, critical deficits were identified. Only 24.3% recognized mechanical ventilation as a risk factor for ventilator‐associated pneumonia (a common HAI), and just 56.7% correctly identified common bacterial agents. A significant knowledge‐practice gap was observed; while knowledge did not differ by academic program, practice scores varied significantly (*p* < 0.001). In‐service training was strongly associated with better practice scores (*p* < 0.001) but not with knowledge. Latent class analysis revealed three distinct profiles: “High Performers” (60.2%), “Motivated Learners” (36.1%) with good practice but lower knowledge, and a “Disengaged” group (3.7%) with poor scores across all domains.

**Conclusion:**

UB allied health students possess foundational HAI knowledge but appear critically underprepared in key clinical areas, with a clear disconnect between theory and practice. This evidence suggests an urgent need to consider a shift from uniform curricula to tailored, competency‐based education integrating robust theory with mandatory simulation and clinical practice. Such a reform could contribute to mitigating Belize’s high HAI burden.

## 1. Introduction

Healthcare‐associated infections (HAIs) represent a critical global public health challenge, significantly contributing to patient morbidity, mortality, and escalating healthcare costs. These infections, defined as those acquired during the course of medical treatment but not present or incubating at the time of admission, arise from exposure to pathogens within healthcare environments and the vulnerabilities associated with invasive procedures and devices [1, 2]. The spectrum of HAIs ranges from localized infections to life‐threatening conditions, directly jeopardizing patient safety by prolonging hospital stays and increasing the risk of fatalities. The burden of HAIs, however, is not uniformly distributed, with low‐ and middle‐income countries (LMICs) bearing a disproportionate weight. Evidence suggests the prevalence in these settings is 5.7–19.1 times higher than in developed nations, highlighting a stark disparity in the capacity to prevent and control these infections [3].

This disparity is acutely felt in the Latin America and Caribbean (LAC) region, where nosocomial infections account for a significant portion of hospitalizations, underscoring systemic challenges in infection control [4, 5]. Within this context, Belize faces a particularly concerning situation. A study estimated that nearly 40% of patients in Belizean hospitals contract an HAI, a rate far exceeding global averages and pointing to profound infrastructural and procedural challenges [6]. Contributing factors, as identified by the Pan American Health Organization [7], include limited resources, inadequate availability of essential equipment like handwashing stations and personal protective gear, and potential gaps in the knowledge and practices of healthcare personnel [8]. The role of healthcare workers is pivotal, as they can either be the first line of defense or, in cases of poor hygiene and inadequate knowledge, potential vectors for cross‐contamination [9].

The foundation for effective infection prevention is laid during the training of future healthcare professionals. The knowledge, attitudes, and practices instilled in allied health students are crucial determinants of future patient safety. While some studies indicate that nursing students may possess a moderate understanding of HAIs, there is a clear consensus on the need for enhanced and standardized education to bridge persistent gaps [10]. For instance, educational interventions have been shown to significantly improve knowledge scores, demonstrating the efficacy of structured training [11]. However, challenges remain, particularly in the consistent application of practical measures such as hand hygiene, needle stick injury prevention, and the correct use of personal protective equipment, which are essential for curtailing the transmission of multidrug‐resistant organisms and blood‐borne pathogens [12–16].

Despite the clear and present danger posed by HAIs in Belize, a critical evidence gap exists regarding the preparedness of the next generation of healthcare workers. There is a complete lack of empirical data on the awareness and knowledge of HAIs among allied health students at the University of Belize (UB). This absence of information hinders the development of targeted educational strategies and curriculum improvements necessary to build a competent workforce capable of mitigating this significant public health threat. Therefore, this study aims to conduct a multidimensional analysis to determine the level of knowledge, attitudes, and preventive practices (KAP) regarding HAIs among future allied health professionals at UB. By identifying gaps and distinct learner profiles, this research provides the essential evidence base to inform targeted curricular reform and clinical training at UB. Such institution‐specific data is a critical first step in developing a national strategy to build a competent, safety‐oriented healthcare workforce capable of addressing Belize’s disproportionate HAI burden.

## 2. Methodology

### 2.1. Study Design and Setting

A descriptive cross‐sectional study was conducted to assess the KAP regarding HAIs among allied health students at the UB. This design was selected, as it is optimal for determining the prevalence of a condition or characteristic at a single point in time, providing a snapshot of the current situation and enabling the identification of associations between variables (Wang and Cheng, 2020). The study was set within the Faculty of Health Sciences at UB, the national UB, a Central American nation with a diverse multicultural population and a developing healthcare system facing unique challenges in infection control [7, 17].

### 2.2. Study Population and Sampling

The target population consisted of all undergraduate students enrolled in the three major allied health programs: Nursing, Pharmacy, and Medical Laboratory Science. The total population size was 444 students, comprising both full‐time and part‐time students.

A simple random sampling technique was employed to ensure each member of the population had an equal probability of selection, thereby minimizing selection bias and enhancing the representativeness of the sample [18]. The sample size was calculated using the standard formula for a finite population. With a 95% confidence level (*Z* = 1.96), a margin of error of 5% (*d* = 0.05), and a population proportion (*p*) of 0.5 to ensure maximum variability, the initial sample size (*n*) was calculated as 384. This was then adjusted for the finite population using the formula na = *n*/[1 + (n/N)], yielding a minimum sample of 206. Accounting for a potential 20% non‐response rate, the final target sample size was set at 247 participants. Of these 247 randomly approached students, 210 completed the questionnaire (85.0% response rate). No missing data were present because the electronic platform required responses to all items before submission. Reasons for nonparticipation were not systematically recorded, but anecdotal reports from students cited time constraints and competing academic obligations. A participant flow diagram is provided in Supp_Figure 6.

### 2.3. Data Collection Instrument and Procedure

#### 2.3.1. Instrument Development and Adaptation

The questionnaire was adapted from the validated instrument by Agarwal and Mohan [11], originally designed for medical students in India, to fit the Belizean context and the specific allied health programs (Nursing, Pharmacy, and Medical Laboratory Science). The adaptation process involved: (1) contextualizing terminology to reflect Belizean healthcare practices and local HAI epidemiology; (2) modifying questions to align with the scope of practice for each allied health profession; (3) expanding the practice section to include profession‐specific infection control procedures; and (4) translating complex medical terms into accessible language appropriate for undergraduate students while maintaining scientific accuracy. A full copy of the adapted questionnaire is provided as a supporting file (Supporting Questionnaire).

### 2.4. Content Validity

Following adaptation, the instrument underwent rigorous content validation by a panel of three independent experts in infection prevention and control (IPC) from the UB’s Faculty of Health Sciences. The expert panel assessed each item for relevance to HAI prevention in the Belizean context, clarity of wording, appropriateness for the target population, and comprehensiveness of coverage across the three domains (knowledge, attitudes, and practices). Experts rated each item on a 4‐point Likert scale (1 = not relevant to 4 = highly relevant), and the content validity index (CVI) was calculated. Item‐level CVI values ranged from 0.83 to 1.00. Items with CVI < 0.78 were revised or removed. The final instrument achieved a scale‐level CVI of 0.91, indicating excellent content validity. Construct validity was not formally assessed (e.g., through factor analysis) due to the small pilot sample size; however, the instrument was adapted from a previously validated KAP questionnaire and underwent expert content validation.

### 2.5. Pilot Testing

The adapted questionnaire was pilot‐tested on a convenience sample of 20 allied health students (8 Nursing, 7 Pharmacy, and 5 Medical Laboratory Science) who were excluded from the main study sample. Pilot participants completed the questionnaire and subsequently participated in cognitive interviews to assess comprehension of each question, interpretation consistency with intended meaning, ease of navigation through the electronic format, and time required for completion (mean completion time = 18 min). Based on pilot feedback, five questions were reworded for clarity, two response options were modified, and the question order was reorganized to improve flow. While the pilot sample of 20 was modest, it exceeded the minimum recommended for instrument pretesting in health professions education research, and reliability estimates were subsequently confirmed in the full study sample.

### 2.6. Reliability Assessment

Following pilot testing, reliability was assessed through two methods:

1. Internal Consistency: Cronbach’s alpha coefficient was calculated for the knowledge scale (7 items) and practice scale (7 items) using pilot data. The knowledge scale demonstrated acceptable internal consistency (*α* = 0.82), and the practice scale showed good internal consistency (*α* = 0.79).
2. Test‐Retest Reliability: A subset of pilot participants (*n* = 15) completed the same questionnaire 2 weeks after initial administration to assess stability over time. Intraclass correlation coefficients (ICC) were calculated for total knowledge scores (ICC = 0.88, 95% CI: 0.79–0.94) and total practice scores (ICC = 0.85, 95% CI: 0.74–0.92), indicating excellent temporal stability.

### 2.7. Final Instrument Structure

Based on the validation process, the final questionnaire comprised four sections:

1. Demographic Characteristics (8 items): Age, gender, program of study, year of enrollment, prior healthcare experience, in‐service training participation, hepatitis B vaccination status, and institutional knowledge monitoring exposure.
2. Knowledge Assessment (7 multiple‐choice questions): Evaluating understanding of HAI definitions (1 item), transmission routes (1 item), causative agents (1 item), types of HAIs (2 items; note that one item asked students to identify mechanical ventilation as a risk factor for ventilator‐associated pneumonia, a common device‐related HAI), prevention methods (1 item), and vaccine‐preventable infections relevant to healthcare workers (1 item). A composite knowledge score (range 0–7) was calculated as the sum of correct responses.
3. Attitude Assessment (4 items): Evaluating students’ perceptions of their professional role in HAI prevention, perceived importance of IPC training, confidence in applying IPC measures, and recognition of personal educational needs. Response options were dichotomous (agree/disagree) due to the constraints of the electronic survey platform, though we acknowledge this as a limitation.
4. Self‐Reported Practices (7 items): Assessing compliance with standard IPC measures including hand hygiene (2 items), personal protective equipment use (2 items), safe injection practices (1 item), environmental cleaning (1 item), and jewelry removal (1 item). A composite practice score (range 0–7) was calculated based on reported adherence to recommended practices.

### 2.8. Final Reliability in Study Sample

Internal consistency for the main study sample (*N* = 210) was reassessed and found to be acceptable for the knowledge scale (Cronbach’s *α* = 0.85) and practice scale (Cronbach’s *α* = 0.81), confirming the instrument’s reliability in the target population.

### 2.9. Data Management and Analysis

All data collected were anonymized at the point of collection, with no personal identifiers recorded. The data were stored in password‐protected digital folders and analyzed using statistical software. Quantitative data from the questionnaires were organized and cleaned in a spreadsheet before being imported into SPSS for advanced statistical analysis.

Data analysis employed both descriptive and inferential statistics. Descriptive statistics (frequencies, percentages, means, and standard deviations) were used to summarize demographic variables, knowledge scores, attitudes, and practices. Inferential analyses included chi‐square tests to examine relationships between categorical variables (e.g., attitude and practice), independent *t*‐tests to compare mean knowledge and practice scores between groups (e.g., by gender or training), and one‐way ANOVA to compare scores across the three academic programs. A multivariable linear regression analysis was performed to identify significant predictors of the knowledge score, controlling for demographic and institutional factors. A *p* value of less than 0.05 was considered statistically significant for all tests. The optimal number of latent classes was determined by comparing model fit indices (Akaike Information Criterion [AIC] and Bayesian Information Criterion [BIC]) and interpretability. The 3‐class solution showed the best fit and good classification certainty (entropy = 0.85).

### 2.10. Ethical Considerations

The study protocol received full approval from the UB Institutional Review Board (04‐26‐24‐53891) prior to commencement. The first section of the electronic questionnaire served as a Participation Information Sheet, detailing the study’s aims, procedures, potential risks, and benefits. Implied consent was deemed appropriate as the study presented minimal risk, participation was entirely voluntary, and consent was documented via the voluntary completion and submission of the anonymous questionnaire. Participants were informed of their right to withdraw from the study at any point without penalty. All data were handled with strict confidentiality, and the study was conducted in accordance with the ethical principles outlined in the Declaration of Helsinki.

## 3. Results

### 3.1. Participant Demographics and Response Rate

**FIGURE 1:**
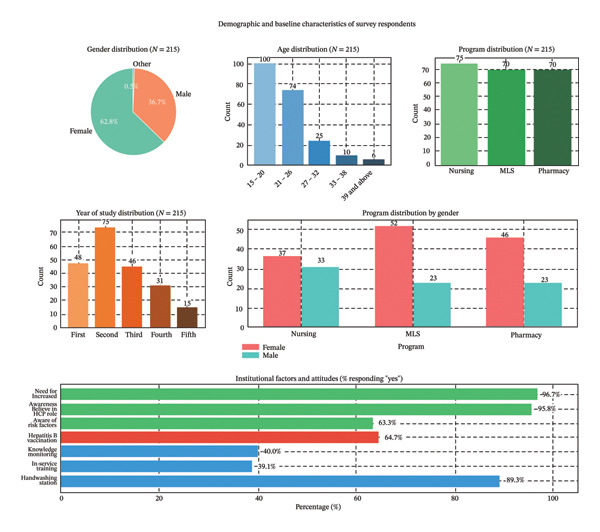
Demographic characteristics.

From a calculated sample size of 247, a total of 210 completed responses were received, yielding a robust response rate of 85.0%. The cohort was predominantly female (61.9%, *n* = 130), reflecting the gender distribution typical of allied health fields. The respondents were relatively evenly distributed across the three academic programs: Nursing (33.8%, *n* = 71), Pharmacy (33.3%, *n* = 70), and Medical Laboratory Science (32.9%, *n* = 69). The majority of participants (44.8%, *n* = 94) were in their second year of study, with fifth‐year students representing the smallest group (2.9%, *n* = 6). The demographic composition of the sample is detailed in Figure 1.

### 3.2. Knowledge of HAIs

**FIGURE 2:**
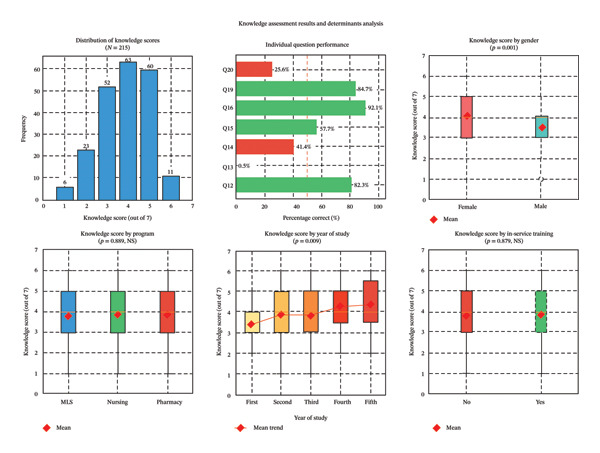
Distribution of knowledge scores.

A composite knowledge score was calculated based on seven key questions, with a maximum possible score of 7. The mean knowledge score across all participants was 3.84 (SD = 1.59), equivalent to 54.9%, indicating a moderate overall understanding of HAIs. The distribution of these scores is visualized in Figure 2.

Analysis revealed significant strengths and critical gaps in specific knowledge domains, detailed in Supp_Figure 1. The vast majority of students demonstrated a strong understanding of HAI transmission routes (81.9% correct) and effective prevention methods, with 83.8% correctly identifying that reusing gloves for different patients is unsafe. Furthermore, 84.3% knew that anemia is not a vaccine‐preventable disease for healthcare personnel. However, profound deficits were uncovered in fundamental areas. A striking 75.6% of students correctly defined a nosocomial infection as one acquired in a hospital, leaving nearly a quarter (24.4%) with a foundational misconception. Knowledge of causative agents was also moderate, with only 56.7% correctly identifying common bacterial agents (*Staphylococcus aureus*, *Pseudomonas aeruginosa*, and *E. coli*). Most critically, when asked to identify a risk factor for HAIs (specifically, that mechanical ventilation is associated with ventilator‐associated pneumonia), only 24.3% selected the correct response. This rate was statistically indistinguishable from those incorrectly selecting “fungal infection” (24.3%) and lower than those choosing “strep throat” (29.5%).

Statistical analysis revealed that knowledge scores were significantly higher among female students (mean = 4.04) compared to males (mean = 3.51, *p* = 0.001). A significant positive trend was also observed with the year of study (*p* = 0.009). In contrast, no significant differences in knowledge were found across the different academic programs (*p* = 0.889), a finding further supported by the multivariate regression model (Supp_Figure 2, Supp_Table 1).

### 3.3. Self‐Reported Preventive Practices

**FIGURE 3:**
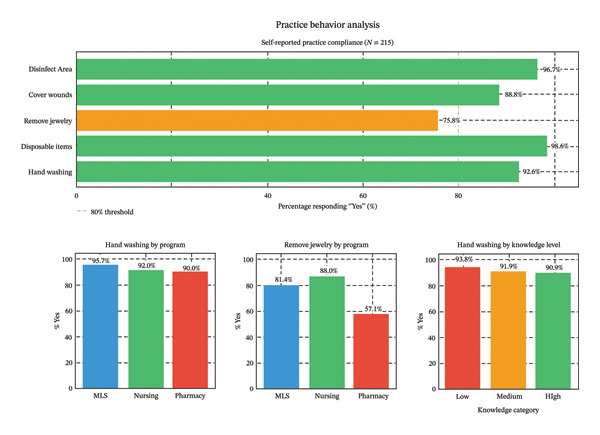
Practice behavior analysis.

Analysis of self‐reported infection control practices revealed generally high self‐reported compliance with fundamental measures. Nearly all students reported using disposable items (98.6%) and practicing hand washing (92.6%). However, self‐reported compliance dropped for more specific practices, such as removing jewelry beforehand for hygiene (75.8%), as shown in Figure 3. Notably, a significant disconnect between knowledge and practice was observed. While knowledge scores did not differ by program, practice scores varied significantly (*p* < 0.001), with a comparative analysis by program presented in Supp_Figure 2. Pharmacy students reported the lowest practice compliance (mean = 4.16), notably in jewelry removal (57.1%), which was significantly lower than that of Medical Laboratory Science (mean = 4.69) and Nursing (mean = 4.72) students. Furthermore, participation in in‐service training had no significant association with knowledge scores (mean with training = 3.85 vs. 3.83 without, *p* = 0.879) but was strongly associated with higher and more consistent self‐reported practice scores (mean = 4.75 with training vs. 4.38 without, *p* < 0.001), as detailed in Supp_Figure 2.

### 3.4. Multivariable and Latent Class Analysis

**FIGURE 4:**
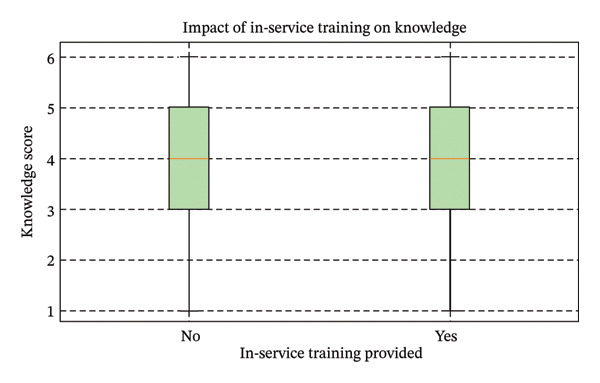
Latent class analysis (clustering approach).

A multivariable regression model was constructed to identify determinants of knowledge scores (Supp_Figure 2, Supp_Table 1). The model confirmed that awareness of HAI risk factors was the strongest positive predictor (*β* = 0.616, *p* < 0.001), while male gender was a significant negative predictor (*β* = −0.473, *p* = 0.002). Academic program, in‐service training, and institutional knowledge monitoring showed no significant association. To further elucidate student profiles, a latent class analysis was performed, identifying three distinct subgroups (Figure 4): (1) High Performers (60.2%): Students with high knowledge (mean = 4.63) and high practice scores (mean = 4.67), strong beliefs, and recognition of educational needs; (2) Motivated Learners (36.1%): Students with lower knowledge (mean = 2.65) but good practice behaviors (mean = 4.33), who recognize their knowledge gaps and the need for education; and (3) Disengaged (3.7%): A small but critical group with low knowledge (mean = 2.13), lower practice (mean = 3.50), weak belief in their professional role (37.5%), and no recognition of the need for further education.

## 4. Discussion

This study provides the first comprehensive, multidimensional analysis of HAI awareness among future allied health professionals in Belize, revealing three core findings: a moderate level of foundational knowledge punctuated by critical clinical gaps (Figure 2), a significant dissociation between theoretical knowledge and self‐reported practice (Figure 3), and the existence of distinct learner profiles with differing educational needs (Figure 4). The findings reveal a complex educational landscape characterized by moderate foundational knowledge alongside critical specific gaps, and most notably, a concerning disconnect between theoretical understanding and practical application—a finding that echoes similar challenges noted in broader healthcare education research [19, 20].

The overall moderate knowledge level (Figure 2) indicates that while students grasp basic principles, their preparation remains inadequate for the nuanced demands of infection prevention in a high‐risk setting like Belize, where HAI prevalence is alarmingly high [6]. This finding aligns with the recognized disparity in HAI burden between LMICs and developed nations, where prevalence can be 5.7 to 19.1 times higher [3]. The moderate knowledge score of 54.9% is consistent with previous research indicating that nursing students generally have a moderate average of knowledge concerning HAIs [10], though it falls considerably below the levels achieved through structured training interventions in other settings [11]. A particularly critical deficit was identified in recognizing device‐associated infection risks. As detailed in Supp_Figure 1, the extremely low recognition that mechanical ventilation is a risk factor for ventilator‐associated pneumonia (24.3% correct) suggests the curriculum may not adequately equip students with the clinical knowledge needed to prevent one of the most common and dangerous device‐related HAIs. This is especially concerning given that ventilator‐associated pneumonia represents one of the most common hospital‐associated infections in Latin American countries [21]. This substantial gap in understanding specific HAI types contrasts with studies showing better recognition of general prevention methods but aligns with research indicating persistent challenges in practical application of infection control knowledge [22, 23].

One of the most instructive findings was the clear dissociation between knowledge and self‐reported practice. While knowledge was consistent across programs, a significant divergence in practice scores was observed (Figure 3, Supp_Figure 4). The notably lower compliance among Pharmacy students, particularly in specific practices like jewelry removal, suggests that program‐specific clinical cultures and the “hidden curriculum” may be more powerful drivers of behavior than formal academic instruction. This knowledge‐practice gap reflects similar challenges noted in previous research where medical students demonstrated adequate knowledge of infection sources but showed room for improvement in practical prevention measures [11]. This disconnect is further supported by the association observed with in‐service training, which was significantly associated with higher self‐reported practice scores (Supp_Figure 4) without a measurable association with knowledge. This finding suggests that hands‐on, practical training may be an important factor associated with translating abstract knowledge into reported behavior. The finding is consistent with educational interventions noted in previous studies [19, 24] and underscores the potential importance of continuous education as a component of infection control programs. This disconnect is powerfully corroborated by the impact of in‐service training, which significantly improved practice scores (Supp_Figure 5) without a measurable effect on knowledge, highlighting that hands‐on, practical training is an essential missing link for translating abstract knowledge into ingrained behavior. This finding supports the effectiveness of educational interventions noted in previous studies [19, 24] and underscores the importance of continuous education as a key component of infection control programs.

The latent class analysis (Figure 4) provides a crucial, person‐centered perspective, crystallizing the student body into three distinct and actionable profiles. The large “Motivated Learners” cohort, characterized by lower knowledge but good practice and recognition of their own educational needs, represents a highly strategic group for targeted interventions; their positive attitudes and self‐awareness present a leverageable asset for bridging knowledge gaps. In contrast, the small but critical “Disengaged” profile presents a potential challenge; such students might act as a reservoir for suboptimal infection control practices if not identified early. This group may require early, targeted remediation to foster professional engagement. This profiling approach offers insights into the varied educational needs within healthcare student populations, complementing previous research that has primarily focused on aggregate knowledge scores [10, 22]. The regression analysis (Supp_Figure 2, Supp_Table 1) further refines our understanding, identifying awareness of risk factors as the strongest cognitive predictor of knowledge. This underscores the importance of comprehensive education that specifically addresses risk factor recognition, as emphasized in infection control guidelines [1, 25]. The negative association with male gender warrants further investigation into underlying social or educational dynamics, particularly given the female‐dominated nature of allied health fields reflected in our demographic data. Crucially, the lack of association with passive institutional factors like knowledge monitoring suggests that merely providing information may be insufficient; active, practical, and engaging training appears more strongly associated with self‐reported competence and compliance. This finding aligns with research emphasizing the need for proper work organization and adequate time for performance improvement in healthcare settings [20], and it supports the call for implementing structured education strategies throughout medical training [26]. In conclusion, this study reveals a future healthcare workforce with a strong ethical foundation but critical gaps in specific knowledge and clinical readiness. The disparities uncovered suggest a pedagogical shift at UB from a model of knowledge transfer to a competency‐based approach, consistent with recommendations for enhancing infection control training in healthcare education [12, 24, 27]. This new model must robustly integrate theoretical teaching with simulated and clinical practical training, deliberately tailored to address the distinct needs of the identified student profiles to ensure a safer healthcare environment for Belize.

### 4.1. Implications for Latin America, the Caribbean, and Small Island Developing States (SIDS)

While the findings are drawn from a single university, the detailed contextual description supports transferability; nevertheless, direct generalization to other settings requires confirmation through multisite studies. The findings from this Belizean study carry important implications that extend beyond national borders, offering insights relevant to the broader LAC region and to SIDS globally. Belize exemplifies the challenges faced by many resource‐limited healthcare systems in the region, where HAI prevalence disproportionately exceeds global averages [3, 7, 28]. The moderate knowledge scores (54.9%) and critical clinical gaps identified—particularly the poor recognition of device‐associated infections—likely reflect similar educational challenges across the LAC region, where standardized IPC curricula remain inconsistently implemented across training institutions [4].

For SIDS specifically, several unique vulnerabilities amplify the urgency of the findings. First, the concentration of tertiary healthcare services in a small number of facilities means that lapses in IPC by even a few healthcare workers can rapidly affect large proportions of the patient population. Second, the frequent rotation of students across limited clinical sites increases the risk of cross‐institutional transmission if IPC practices are not consistently reinforced. Third, many SIDS, including Belize, rely on healthcare professionals trained abroad or in varied educational systems, potentially introducing inconsistent IPC knowledge and practices into the workforce [29]. Fourth, the small size of the healthcare workforce in SIDS limits the availability of dedicated IPC specialists, placing greater responsibility on frontline generalists—precisely the allied health professionals in training who were the focus of this study.

The distinct learner profiles identified through latent class analysis—High Performers (60.2%), Motivated Learners (36.1%), and Disengaged (3.7%)—likely exist across LAC training institutions, though their proportions may vary based on local educational contexts. The substantial “Motivated Learners” cohort, characterized by good practice but lower knowledge, represents a particularly strategic target for region‐wide educational interventions; these students recognize their own gaps and are primed for structured learning opportunities. Conversely, the small “Disengaged” minority, if present across the region, could disproportionately impact patient safety and warrants early identification and remediation strategies in every LAC country’s educational framework.

The dissociation between knowledge and practice observed in this study—and the significant improvement in practice associated with in‐service training despite no corresponding knowledge gain—carries particular resonance for SIDS. This finding suggests that hands‐on, practical training may compensate for uneven theoretical preparation, offering a pragmatic approach for resource‐constrained settings where comprehensive curriculum reform may be slow. Regional bodies such as PAHO and the Caribbean Public Health Agency (CARPHA) could leverage this insight to promote standardized simulation‐based IPC training modules that can be implemented across multiple institutions without requiring extensive infrastructure investment.

Furthermore, the strong association between awareness of risk factors and overall knowledge (*β* = 0.616, *p* < 0.001) underscores the importance of integrating risk factor recognition into IPC curricula across the region. Given the high prevalence of device‐associated infections in LAC hospitals [4, 6], the critical deficit in recognizing mechanical ventilation as a risk factor for HAI type (only 24.3% correct) likely represents a region‐wide knowledge gap requiring urgent attention in medical and allied health training programs throughout Latin America and the Caribbean.

Finally, the gender differences observed (higher knowledge among female students) warrant exploration across LAC contexts. The female‐dominated nature of allied health fields in the region may influence educational dynamics, peer learning, and professional socialization in ways that could be leveraged to strengthen IPC training for all students. Cross‐country comparative research examining gender, educational outcomes, and IPC compliance would help determine whether these patterns are consistent across the region and what interventions might address disparities.

In summary, this Belizean case study serves as a microcosm of broader regional challenges in HAI prevention education. The findings call for a coordinated LAC and SIDS response: harmonized IPC competency standards, regionally validated assessment tools, shared simulation resources, and faculty development programs that build capacity for practical IPC training across all allied health disciplines. Such regional collaboration is essential for cultivating a healthcare workforce across Latin America and the Caribbean that is truly prepared to combat the disproportionate burden of HAIs.

### 4.2. Recommendations

#### 4.2.1. Replace the Entire Recommendations Section With the Following Condensed and Directly Linked Version

Based on the findings of this study, several strategic recommendations are proposed to enhance HAI preparedness among future healthcare professionals at the UB. Each recommendation is directly linked to a specific result from the study.

First, given that only 24.3% of students recognized mechanical ventilation as a risk factor for ventilator‐associated pneumonia and only 56.7% correctly identified common bacterial agents, it is recommended that the core curriculum of all allied health programs at UB integrate specific teaching on device‐associated infections (including ventilator‐associated pneumonia, central line‐associated bloodstream infections, and catheter‐associated urinary tract infections) and on the microbiology of common HAI pathogens.

Second, because practice scores varied significantly by program (*p* < 0.001) despite no difference in knowledge, and because pharmacy students reported lower compliance with jewelry removal (57.1% versus 72%–81% in other programs), educational interventions should be tailored to address program‐specific behavioral patterns. The distinct learner profiles identified through latent class analysis (High Performers 60.2%, Motivated Learners 36.1%, and Disengaged 3.7%) further support a tailored approach. Motivated Learners, who demonstrate good practice but lower knowledge, may benefit from targeted knowledge‐reinforcement modules. The small Disengaged group may require early identification, mentorship, and remediation.

Third, given that in‐service training was strongly associated with higher self‐reported practice scores (mean 4.75 with training versus 4.38 without, *p* < 0.001) but not with knowledge scores, the university should consider formalizing in‐service training during clinical rotations. Such practical training may help bridge the observed dissociation between theoretical knowledge and reported practice.

Fourth, future research should be conducted longitudinally to track the evolution of knowledge and practices and expanded to include other healthcare training institutions in Belize as they mature. Future qualitative research (e.g., focus groups) is recommended to explore the contextual and cultural factors underlying program‐specific practice differences, such as the lower reported jewelry removal among pharmacy students.

Based on the specific findings, a competency‐based syllabus framework for allied health education at UB is proposed. This framework includes a foundational component (Years 1–2) covering HAI epidemiology, microbiology, and standard precautions; a clinical skills component (Years 2–3) with mandatory simulation‐based training (minimum 20 h) and Objective Structured Clinical Examinations focusing on device‐associated infection prevention; a clinical immersion component (Years 3–4) with structured practicums, case‐based learning using local HAI incidents, and student‐led quality improvement projects; and a capstone component (Year 4) with interprofessional education modules across nursing, pharmacy, and medical laboratory science. Detailed implementation strategies (vertical and horizontal integration, competency‐based assessment, clinical partnership models, and continuous quality improvement) would require curriculum committee consultation and are beyond the scope of this study. This framework directly responds to the three learner profiles: High Performers would be challenged through advanced case studies; Motivated Learners would benefit from structured simulation to bridge the knowledge‐practice gap; and the Disengaged cohort would receive targeted mentorship and early remediation.

### 4.3. Limitations

While this study provides valuable insights, its findings must be interpreted considering several limitations. The cross‐sectional design offers a snapshot in time and cannot establish causal relationships between education, knowledge, and practices; all associations reported should be interpreted as correlational.

The reliance on self‐reported practices is susceptible to social desirability bias, where participants may overstate their adherence to ideal protocols. The very high self‐reported compliance rates for certain practices (e.g., 98.6% for disposable item use, 92.6% for hand washing) substantially exceed observed hand hygiene compliance rates reported in observational studies of healthcare workers (typically 40%–60%), suggesting that social desirability bias likely inflated the reported figures. Therefore, the absolute practice scores probably overestimate true infection control behaviors. However, the relative differences between programs (e.g., pharmacy students’ lower reported jewelry removal) and the associations with in‐service training are less susceptible to this bias and remain valid for comparative purposes.

The study was conducted at the UB, which is the only national university in Belize offering degree programs in Pharmacy, Nursing (BSN level), and Medical Laboratory Science. Thus, there are no other comparable institutions with allied health students at equivalent stages of professional preparation. While this limits statistical generalization to other countries, the detailed contextual information provided (e.g., Belize’s HAI burden from Ugalde and Guzman [6]; PAHO country profile [7]) supports transferability of findings to other SIDS and resource‐limited settings in Latin America and the Caribbean.

Furthermore, the instrument used to assess attitudes was limited to only four dichotomous (agree/disagree) items. This binary response format substantially restricts response variability, prevents detection of nuanced or ambivalent attitudes, and precludes calculation of internal consistency for the attitude domain. While the dichotomous format was necessitated by constraints of the electronic survey platform, future studies should employ multipoint Likert scales (e.g., 5 point or 7 point) to capture gradations in student perceptions of professional responsibility, confidence, and educational needs. Consequently, the attitude findings should be interpreted as preliminary indicators rather than definitive measures of student dispositions.

The timing of the research within the academic calendar may have influenced participation and response quality. Finally, the study did not account for all potential confounding variables, such as the specific clinical experiences of each student prior to the survey, which could influence both their knowledge and reported practices.

Despite these limitations, this study offers the first empirical evidence on HAI KAP among allied health students in Belize, utilizing a robust sampling method, a high response rate (85%), and advanced analytical techniques like latent class analysis to provide nuanced, actionable insights for curriculum development.

## 5. Conclusion

This study illuminates the critical state of HAI awareness among allied health students at the UB, revealing a workforce in training that possesses a foundational understanding of infection control but appears hampered by significant specific knowledge gaps and a troubling disconnect between what is known and what is self‐reported as practiced. The identification of a moderate overall knowledge score (54.9%), coupled with a profound deficit in recognizing device‐associated infection risks (only 24.3% recognized mechanical ventilation as a risk factor for ventilator‐associated pneumonia), suggests an urgent need for curriculum reform. The discovery of distinct student profiles, from the High Performer to the Disengaged, indicates that a one‐size‐fits‐all educational approach may be insufficient. The path forward likely requires a concerted effort to transform HAI education from a theoretical exercise into a competency‐based model deeply integrated with practical, hands‐on training. Based on the findings, a competency‐based syllabus framework for allied health education at UB is proposed, including foundational courses in HAI epidemiology and microbiology (Years 1–2), simulation‐based training with Objective Structured Clinical Examinations (Years 2–3), structured clinical practicums with case‐based learning (Years 3–4), and interprofessional education capstone modules (Year 4). This framework directly addresses the learner profiles identified: High Performers would be challenged through advanced case studies; Motivated Learners would benefit from structured simulation bridging the knowledge‐practice gap; and the Disengaged cohort would receive targeted mentorship and early remediation. If implemented, such a competency‐based curriculum could systematically address the identified gaps, helping to ensure that graduates enter the workforce not merely knowledgeable about HAIs but demonstrably competent in preventing them. This strategic educational reform could contribute to building a more proficient healthcare workforce in Belize and ultimately improving patient safety.

## Funding

The authors did not receive support from any organization for the submitted work.

## Ethics Statement

Ethics approval was received from the University of Belize IRB (04‐26‐24‐53891).

## Consent

Verbal/electronic consent was sought and received before data collection.

## Conflicts of Interest

The authors declare no conflicts of interest.

## Supporting Information

Additional supporting information can be found online in the Supporting Information section.

## Supporting information


**Supporting Information** The supporting information file (SUPPLEMENTARY_HAIs_Preparing the Frontline.docx) contains additional figures and tables referenced throughout the manuscript, including: Supp_Figure 1: Descriptive breakdown of knowledge scores across specific domains. Supp_Figure 2: Multivariable regression model identifying determinants of HAI knowledge scores. Supp_Figure 3: Attitude‐practice gap analysis. Supp_Figure 4: Comparative analysis of knowledge and practice scores by academic program. Supp_Figure 5: Impact of institutional factors on knowledge and practice scores. Supp_Figure 6: Participant flow diagram showing sample selection and response rate. Supp_Table 1: Detailed results of the regression analysis with coefficients, confidence intervals, and significance levels. Supporting Questionnaire: Full text of the adapted KAP instrument. These materials provide expanded data visualizations and statistical details that support the findings reported in the main text.

## Data Availability

All the data generated and associated with this research has been provided in this article.
