# Supplementary material for: Preparing the Frontline: Profiling Knowledge, Attitudes, and Practice Gaps in Healthcare‐Associated Infection Prevention Among Future Health Professionals in Belize
Source: Interdiscip Perspect Infect Dis. 2026 Jun 22;2026:3745039. doi: 10.1155/ipid/3745039 (PMC13287823; doi:10.1155/ipid/3745039)
Supplement: Supplementary file 1 — Supporting Information The supporting information file (SUPPLEMENTARY_HAIs_Preparing the Frontline.docx) contains additional figures and tables referenced throughout the manuscript, including: Supp_Figure 1: Descriptive breakdown of knowledge scores across specific domains. Supp_Figure 2: Multivariable regression model identifying determinants of HAI knowledge scores. Supp_Figure 3: Attitude‐practice gap analysis. Supp_Figure 4: Comparative analysis of knowledge and practice scores by academic program. Supp_Figure 5: Impact of institutional factors on knowledge and practice scores. Supp_Figure 6: Participant flow diagram showing sample selection and response rate. Supp_Table 1: Detailed results of the regression analysis with coefficients, confidence intervals, and significance levels. Supporting Questionnaire: Full text of the adapted KAP instrument. These materials provide expanded data visualizations and statistical details that support the findings reported in the main text. [file IPID-2026-3745039-s001.docx]

**Preparing the Frontline: Profiling Knowledge, Attitudes, and Practice Gaps in Healthcare-Associated Infection Prevention Among Future Health Professionals in Belize**

Danladi C. Husaini^1,2*^, Sanjana Punjabi^1^, Tatyana Guild^1^, Elodia Solis^1^, and Yusuf Abubakar^1^

**SUPPLEMENTARY RESULTS**

**Supp_Figure 1. Knowledge Score Descriptive**

This bar chart displays the distribution of composite knowledge scores (range 0 to 7) among the 210 allied health students. The mean score was 3.84 (SD 1.59), equivalent to 54.9%, indicating a moderate overall understanding of healthcare‑associated infections.

**Supp_Figure 2. Multivariable Regression for Knowledge Score Determinants**

This forest plot presents the coefficients and 95% confidence intervals from the multivariable linear regression model examining predictors of HAI knowledge scores. Awareness of risk factors (β=0.616, p<0.001) and male gender (β=‑0.473, p=0.002) were significant predictors, whereas academic program and in‑service training were not.

**Supp_Figure 3. Attitude-Practice Gap Analysis**

This figure compares student attitudes (belief in professional role, perceived importance of training, confidence, and recognition of educational needs) with self‑reported practice scores across the three academic programs. The analysis reveals that positive attitudes did not always correspond to higher practice compliance.

**Supp_Figure 4. Comparative Analysis by Program**

This panel of bar charts compares mean knowledge scores and mean self‑reported practice scores among Nursing, Pharmacy, and Medical Laboratory Science students. While knowledge scores did not differ significantly across programs (p=0.889), practice scores varied significantly (p<0.001), with Pharmacy students reporting the lowest compliance.

**Supp_Figure 5. Institutional Factors Impact**

This figure illustrates the association between institutional factors (in‑service training participation, knowledge monitoring, and hepatitis B vaccination status) and mean knowledge and practice scores. In‑service training was strongly associated with higher practice scores (p<0.001) but not with knowledge scores.

**Supp_Figure 6. Participant flow Diagram**

This STROBE‑compliant flow diagram shows the selection of the study sample. Of the 444 total allied health students in the target population, 247 were randomly approached. A total of 210 completed the questionnaire, yielding an 85.0% response rate. No participants were excluded, and no missing data were present due to the electronic platform’s required‑response format.

**Supp_Table 1. Regression Analysis Results**

| Category | Variable | Coefficient | Std Error | p-value | 95% CI Lower | 95% CI Upper | Significance |
| --- | --- | --- | --- | --- | --- | --- | --- |
| **Baseline** | const | 5.258 | 0.664 | <0.001 | 3.950 | 6.567 | *** |
| **Academic Program** | program_Nursing | -0.208 | 0.194 | 0.284 | -0.590 | 0.174 |  |
|  | program_Pharmacy | -0.246 | 0.221 | 0.267 | -0.681 | 0.190 |  |
| **Year in Program** | Year_First | -2.068 | 0.667 | 0.002 | -3.384 | -0.753 | ** |
|  | year_Fourth | -1.142 | 0.695 | 0.102 | -2.512 | 0.229 |  |
|  | year_Second | -1.866 | 0.669 | 0.006 | -3.186 | -0.546 | ** |
|  | year_Third | -2.652 | 0.746 | <0.001 | -4.123 | -1.180 | *** |
|  | year_Fifth | -1.457 | 0.690 | 0.036 | -2.818 | -0.095 | * |
|  | year_First | -1.857 | 0.662 | 0.006 | -3.163 | -0.551 | ** |
|  | year_Fourth | -1.445 | 0.663 | 0.030 | -2.752 | -0.138 | * |
|  | year_Second | -1.476 | 0.645 | 0.023 | -2.747 | -0.205 | * |
|  | year_Third | -1.426 | 0.638 | 0.027 | -2.684 | -0.168 | * |
| **Demographics** | gender_Male | -0.473 | 0.153 | 0.002 | -0.775 | -0.170 | ** |
|  | gender_Other | -0.758 | 1.083 | 0.485 | -2.894 | 1.378 |  |
| **Interventions & Awareness** | in_service_training_Yes | -0.052 | 0.193 | 0.790 | -0.432 | 0.329 |  |
|  | knowledge_monitoring_Yes | -0.069 | 0.173 | 0.690 | -0.410 | 0.272 |  |
|  | hep_b_vaccinated_Yes | 0.290 | 0.159 | 0.070 | -0.024 | 0.604 | † |
|  | aware_risk_factors_Yes | 0.616 | 0.162 | <0.001 | 0.297 | 0.934 | *** |

**Significance Codes:** *** p<0.001, ** p<0.01, * p<0.05, † p<0.10

This table provides the full results of the multivariable linear regression analysis for HAI knowledge scores. For each variable (academic program, year of study, gender, in‑service training, knowledge monitoring, hepatitis B vaccination, and awareness of risk factors), the table reports the coefficient, standard error, p‑value, 95% confidence interval, and significance level. Awareness of risk factors (β=0.616, p<0.001) and male gender (β=‑0.473, p=0.002) were the strongest predictors.

Supplementary_Questionnaire

Questionnaire on Hospital-Acquired Infections Among Allied Health Students

# Introduction

This questionnaire is designed to collect information on allied health students’ socio-demographic characteristics, organizational factors, knowledge, awareness, and self-reported practices related to the prevention of hospital-acquired infections (HAIs). Participation is voluntary, and respondents may decline to answer any question or withdraw at any time. Responses should be provided honestly and will be treated confidentially for research purposes. No personal names or identifying information are required in this questionnaire.

# Participant Consent

By proceeding with this questionnaire, the respondent confirms that the purpose of the study has been explained, that participation is voluntary, and that the information provided will be used only for research purposes.

**Consent statement:** I voluntarily agree to participate in this study.

Response: ________________________________________________

# Questionnaire Items

## Section 1: Socio-Demographic Characteristics

**Instruction:** Please select the most appropriate response for each item.

| **No.** | **Question** | **Response Options** |
| --- | --- | --- |
| 1 | Gender | A. Female B. Male C. Other |
| 2 | Age | A. 15–20 B. 21–26 C. 27–32 D. 33–38 E. 39 and above |
| 3 | In what program are you enrolled? | A. Medical Laboratory Science B. Nursing C. Pharmacy |
| 4 | What year are you in your studies? | A. First B. Second C. Third D. Fourth E. Fifth |

## Section 2: Organizational Factors Influencing HAI Prevention Knowledge and Practices

**Instruction:** Please select the most appropriate response for each item.

| **No.** | **Question** | **Response Options** |
| --- | --- | --- |
| 5 | Is there a hand washing station with soap, running water, and disposable towels? | A. No B. Yes |
| 6 | Does your institution provide in-service training related to infection prevention and control? | A. No B. Yes |
| 7 | Is knowledge of infection prevention and control among staff monitored in the university by management? | A. No B. Yes |
| 8 | I am vaccinated against the Hepatitis B virus. | A. No B. Yes |

## Section 3: Knowledge of Hospital-Acquired Infection Prevention

**Instruction:** Please select the most appropriate response for each item.

| **No.** | **Question** | **Response Options** |
| --- | --- | --- |
| 9 | Are you aware of the risk factors contributing to the spread of HAIs in healthcare settings? | A. Yes B. No |
| 10 | Do you believe healthcare professionals play a significant role in preventing and controlling HAIs? | A. Yes B. No |
| 11 | Do you think there is a need for increased awareness and education about HAIs among allied health students? | A. Yes B. No |
| 12 | Hospital-acquired infections can be transmitted from: | A. Staff to patients B. Patients to staff C. All the above D. None of the above |
| 13 | Nosocomial infections are infections that patients: | A. Acquire from home before reporting to the hospital B. Acquire from the hospital C. Are not at risk of acquiring |
| 14 | Most nosocomial infections are caused by: | A. Multi-drug resistant bacteria B. Multi-drug sensitive bacteria C. Virus D. None of the above |
| 15 | The most likely agent(s) to cause a nosocomial infection include: | A. Staphylococcus aureus B. Pseudomonas aeruginosa C. E. coli D. All of the above |
| 16 | The following are effective ways of preventing hospital-acquired infections except: | A. Frequent hand washing with soap under running water B. Using the same gloves for different patients C. Disinfection and sterilization D. Disposing of used needles in sharp boxes |
| 18 | Choose the option that is not a sterilization process. | A. Steam sterilization using autoclave B. Chemical sterilization using chemicals like glutaraldehyde C. Physical sterilization using distilled water |
| 19 | Personal protective equipment (PPE) does not create a barrier between viruses, bacteria, or fungi and healthcare workers. | A. True B. False |
| 20 | Vaccination decreases hospital-acquired infections. The following are vaccinations for healthcare personnel, except: | A. Influenza B. Hepatitis B C. Anemia D. Meningococcal |
| 21 | One of the types of health-acquired infections encountered in a hospital setting is: | A. Fungal infection B. Nasal congestion C. Mechanical ventilation D. Strep throat |

## Section 4: Self-Reported Practices Towards HAI Prevention

**Instruction:** Please select the most appropriate response for each item.

| **No.** | **Question** | **Response Options** |
| --- | --- | --- |
| 22 | I always wash my hands with soap under running water. | A. Yes B. No |
| 23 | Disposable items, such as gloves, masks, etc., are used once and discarded. | A. Yes B. No |
| 24 | Before beginning hand hygiene, I remove my ring(s), watch, or bracelet. | A. Yes B. No |
| 25 | I cover wounds and cuts on my skin before I start my work. | A. Yes B. No |
| 26 | I often disinfect the working area and ensure that all instruments are decontaminated after each use. | A. Yes B. No |

# Conclusion

Thank you for completing this questionnaire. The information provided will support the assessment of knowledge, awareness, and practices related to hospital-acquired infection prevention among allied health students. Your responses are important for identifying areas where infection prevention education and practice can be strengthened.
